# Molecular Dynamics Study of Zn(Aβ) and Zn(Aβ)_2_

**DOI:** 10.1371/journal.pone.0070681

**Published:** 2013-09-27

**Authors:** Lurong Pan, James C. Patterson

**Affiliations:** Department of Chemistry, University of Alabama at Birmingham, Birmingham, Alabama, United States of America; University of Akron, United States of America

## Abstract

The aggregation of Aβ-peptide (Aβ) is widely considered to be the critical step in the pathology of Alzheimer's disease. Small, soluble Aβ oligomers have been shown to be more neurotoxic than large, insoluble aggregates and fibrils. Recent studies suggest that biometal ions, including Zn(II), may play an important role in the aggregation process. Experimentally determining the details of the binding process is complicated by the kinetic lability of zinc. To study the dynamic nature of the zinc-bound Aβ complexes and the potential mechanisms by which Zn(II) affects Aβ oligomerization we have performed atomistic molecular dynamics (MD) simulations of Zn(Aβ) and Zn(Aβ)_2_. The models were based on NMR data and predicted coordination environments from previous density functional theory calculations. When modeled as 4-coordinate covalently bound Zn(Aβ)*_n_* complexes (where *n* = 1 or 2), zinc imposes conformational changes in the surrounding Aβ residues. Moreover, zinc reduces the helix content and increases the random coil content of the full peptide. Although zinc binds at the N-terminus of Aβ, β-sheet formation is observed exclusively at the C-terminus in the Zn(Aβ) and most of the Zn(Aβ)_2_ complexes. Furthermore, initial binding to zinc promotes the formation of intra-chain salt-bridges, while subsequent dissociation promotes the formation of inter-chain salt-bridges. These results suggest that Zn-binding to Aβ accelerates the aggregation of Aβ by unfolding the helical structure in Aβ peptide and stabilizing the formation of vital salt-bridges within and between Aβ peptides.

## Introduction

Alzheimer's disease is the most common neurodegenerative disorder, impacting more than 35 million people worldwide [1]. The aggregation of amyloid β-peptide (Aβ) is the major pathological event that takes place in Alzheimer's disease [2]. The pathologically relevant Aβ fragments are Aβ(1-40) and Aβ(1-42) with 40 or 42 residues respectively: DAEFRHDSGYEVHHQKLVFFAEDVGSNKGAIIGLMVGGVVIA. Both can form soluble monomers, oligomers, insoluble tangles and amyloid plaques. Aβ(1-42) is more prone to aggregation than Aβ(1-40) [3]. Although Aβ(1-40) is the predominant form of Aβ produced, Aβ(1-42) is the major component of amyloid deposits in senile plaques [4]. Also Aβ(1-42) promotes amyloid deposition but Aβ(1-40) inhibits the process [5]. The soluble forms of Aβ(1-42) are the most toxic species that cause neuronal damage in the brains of Alzheimer's disease patients [6]. Although the detailed mechanism of Aβ aggregation is not known, the amyloid cascade hypothesis suggests that the pathology of Alzheimer's disease arises due to an imbalance between the production and clearance of Aβ [7]. Moreover, the small soluble oligomers of Aβ are allegedly the toxic species and not the amyloid fibrils [8].

Physiological examinations have revealed that disrupted biometal homeostasis is an indication of Alzheimer's disease progression [9]. Tissue analysis showed that the concentrations of transition metal ions in neuropil (normal vs. Alzheimer's disease) are: Zn^2+^ (346 µM vs. 786 µM), Cu^2+^ (69 µM vs. 304 µM) and Fe^3+^ (338 µM vs. 695 µM) [10]. Also, the most recent quantitative analysis in the brain tissue of Alzheimer's disease patients revealed that, compared with the surrounding tissue, concentrations of Fe^3+^, Cu^2+^ and Zn^2+^ in the amyloid deposits are greater by factors of 2, 2.6 and 2.5 respectively [11]. Moreover, *in vitro* experiments have shown that Aβ binds to these metals with relatively high affinity and metal ions generally promote the aggregation of Aβ [12], [13]. Furthermore, metal chelators have been shown to dissolve the proteinaceous deposits from the postmortem brain tissues of people who suffered from Alzheimer's disease [14], [15].

Of the three transition metals that have increased concentrations in Alzheimer's disease, zinc has a much higher extracellular concentration (up to 200 µM) [11] and it has been found at up to 1 mM concentration in amyloid plaques. Zn(II) is labile with a rapid ligand exchange rate in general. Time-resolved spectroscopic and structural experiments suggest that Zn(II) promotes aggregation more strongly through intermolecular bridges forming Zn(Aβ)_2_ species [16]. Studies have revealed that the residues in Aβ that coordinate to Zn(II) are found at the N-terminus, Aβ(1-16), including Asp1, His6, Glu11, His13 and His14 [17]–[35]. However, the β-sheet components of Aβ fibrils were found on the C-terminus, Aβ(17-42) [36]. To date, there is no available X-ray crystal structure for zinc-bound oligomers. However, there is an NMR solution structure (PDB: 1ZE9) [20] for the monomeric Zn-Aβ(1-16) complex, in which the peptide coordinates were obtained from NMR measurements and the zinc coordinates were found from molecular modeling [20]. In addition, NMR structures for monomeric Aβ (PDB: 1Z0Q) [37] and Aβ fibril (PDB: 2BEG) [38] are available.

In this work, molecular dynamics simulations were performed on structures of both Zn(Aβ) and Zn(Aβ)_2_ with different combinations of coordinating residues including the three N-terminal histidines (His6, 13, 14) and the glutamic acid (Glu11) generated from previous quantum mechanics calculations and NMR results (PDB: 1Z0Q) [37]. Our molecular dynamics simulations show how zinc binding affects the secondary structure of Aβ in the N-terminus and influences the overall conformation of the peptide. Also, our simulations show how peptide-peptide interactions promote changes in secondary structure in the presence of zinc and after the loss of zinc.

## Methods

### QM calculations on Zn(Aβ)*n* (where *n* = 1 or 2) complexes

For Zn(Aβ) complexes there is one available NMR solution structure (PDB: 1ZE9) [20], to which we can compare our geometric parameters. In this structure, Zn^2+^ binds to four ligands including three histidines (His6, 13 and 14) and one glutamic acid (Glu11). In molecular dynamics simulations using classical force-fields, metal ions are often treated as free ions that are only “bound” via electrostatic interactions. This method gives all coordination complexes octahedral geometry, which does not match zinc's coordination sphere found from the NMR result for Zn(Aβ). Statistics have shown that though other coordination numbers and geometries have been observed, zinc's coordination environment is most often a slightly distorted tetrahedral four-coordinate complex when bound to proteins [39]–[41]. In our study, the force field parameters for the Zn-peptide bonds were calculated from density functional theory (DFT) [42] calculations on model complexes including [Zn(Im)_3_(Ac)]^+^, Zn(Im)_2_(Ac)_2_, and Zn(Im)_4_ (where Im = imidazole and Ac = acetate). Geometry optimizations were performed using the popular B3LYP [43], [44] density functional with the 6-31+G* [45], [46] basis set. All DFT calculations were performed with Gaussian 09 [47]. Force constants for the Zn coordination sphere in each structure were generated from the Hessian matrix obtained from vibrational frequency analysis and were added to the CHARMM 22 force field with CMAP corrections (CHARMM 22/CMAP) [48] (Table 1) for the molecular dynamics simulations.

**Table 1:** Percentage of Time the Asp23-Lys28 Salt Bridge Is Intact.

Structures	Inter-chain	Intra-chain
Zn(Aβ)	N/A	48.1
Control (Aβ)	N/A	0.5
Zn(Aβ)_2_ at His6 Glu11	0.0	46.7
Control (Aβ)_2_	0.0	33.3
Zn(Aβ)_2_ at Glu11 His13	0.0	44.8
Control (Aβ)_2_	24.5	0
Zn(Aβ)_2_ at Glu11 His14	0	33.7
Control (Aβ)_2_	31.8	5.8
Zn(Aβ)_2_ at His13 His14	0.0	0.0
Control (Aβ)_2_	0.0	26.6

The data show the percentage of time after equilibration that the Asp23-Lys28 salt bridge is intact in each set of simulations. The salt bridge is defined as intact when the O(Asp23)-N(Lys28) distance is ≤4 Å. All results are the average values of three runs for each simulation. Inter- and intra-chain salt bridge percentages were measured separately.

### MD simulations on Zn(Aβ)*n* (where *n* = 1 or 2) complexes

Aβ(1-42) (PDB: 1Z0Q) [37] models were created for all molecular dynamics simulations. In general, the initial structures for Zn(Aβ)*_n_* (where *n* = 1 or 2) complexes were created by using the experimental data and our aforementioned DFT calculations. The initial structure for Zn(Aβ) was created by using the NMR structure for Zn(Aβ(1-16)) (PDB: 1ZE9) [20] as the N-terminus and combining it with residues 17-42 from Aβ(1-42) (PDB: 1Z0Q) [37]. The Zn(II) was covalently bound to His6, 13, 14 and Glu11 as found by NMR, using the force-constants obtained from our DFT calculations. For the Zn(Aβ)_2_ complexes, quantum mechanics calculations revealed that the ligand combinations (2 Glu and 2 His) and 4 His are relatively stable dimeric cross linkages (at pH 7.0). Thus, there are six different possible dimeric structures (Glu11/His6, Glu11/His13, Glu11/His14, His6/His13, His6/His14 and His13/His14). To model the His6/His13 and His6/His14 combinations would have required changing the backbone from what was observed in the NMR. Therefore, the His6/His13 and His6/His14 combinations were not included in this study. Control models were built by removing the Zn(II) from the Zn-bound complexes, thereby keeping the same initial structures (prior to minimization) for the peptides. In this manner, it is possible to show how zinc's effect on the peptide can persist even after dissociation. Ten models were built and simulated. We used all-atom simulations with explicit water solvent (TIP3P) for the systems with our augmented CHARMM 22/CMAP force field (Table S1). We performed conjugate gradient energy minimization on the solvated system, followed by 1.5 ns simulation during which thermal energy was added to the systems gradually, which increased the temperature from 0 to 300 K. Next, we performed 200 ns simulations in triplicate for each complex at constant temperature and pressure (NPT, 1 atm, 300 K) using the program NAMD 2.7 [49]. All data analysis was done using VMD 1.9 [50].

Root mean square deviations (RMSD) of the Cα atoms were measured (Figure S1) for all simulations to evaluate the extent of equilibration. To compare the flexibilities of different regions of Aβ, root mean square fluctuations (RMSF) were measured (Figure S2) for the Cα atoms after the simulations equilibrated. Secondary structure changes over time were measured (using the STRIDE plugin as implemented in VMD [50]) for all peptide species in terms of total percentage secondary structure composition within each peptide chain. Also, we measured the percentage of time each residue exhibited either helix or β-sheet structures after equilibration. The radius of gyration was measured over time in each simulation to determine how binding to zinc affects how compact the peptides are. Salt bridges (Table 1) over time were measured to reveal certain intra-chain and inter-chain interactions.

## Results

**Figure 1:**
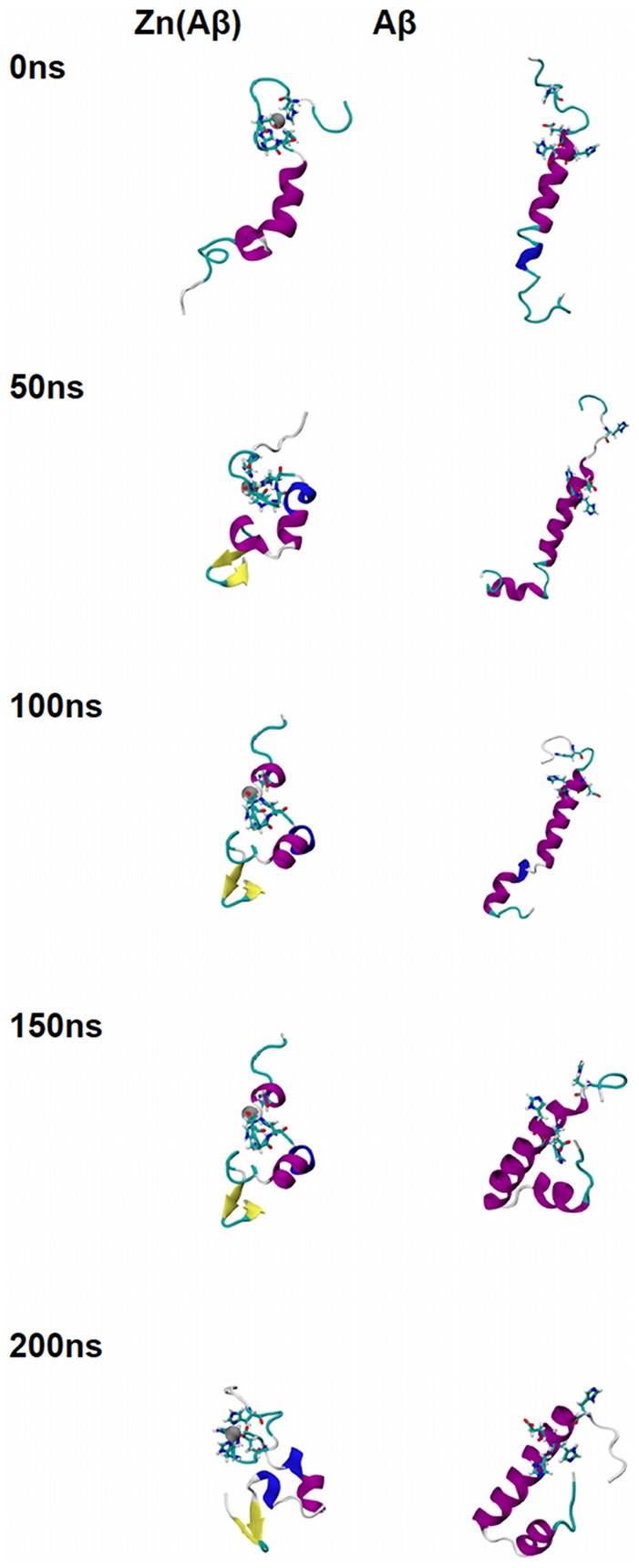
Trajectories of Simulations of Zn(Aβ) and Control. Structures of Zn(Aβ) (where Zn binds at Glu11, His6, 13 14) and the control (Aβ) at 0 ns, 50 ns, 100 ns, 150 ns and 200 ns are shown. The peptide has the same initial structure in the Zn(Aβ) complex and the control simulation. Gray spheres are zinc atoms. Zn-binding residues including Glu11, His6, 13, 14 are shown in licorice, where red, blue and cyan colors shows the atoms and bonds for oxygen, nitrogen and carbon respectively. Peptide backbones are shown in cartoon style where cyan indicates turn, white is coil, purple is alpha-helix, blue is 3_10_ helix, and yellow is β-sheet. All simulations were performed in explicit water. Water molecules are not shown for clarity. Each is peptide oriented with the N-terminus at the top and C-terminus towards the bottom.

**Figure 2:**
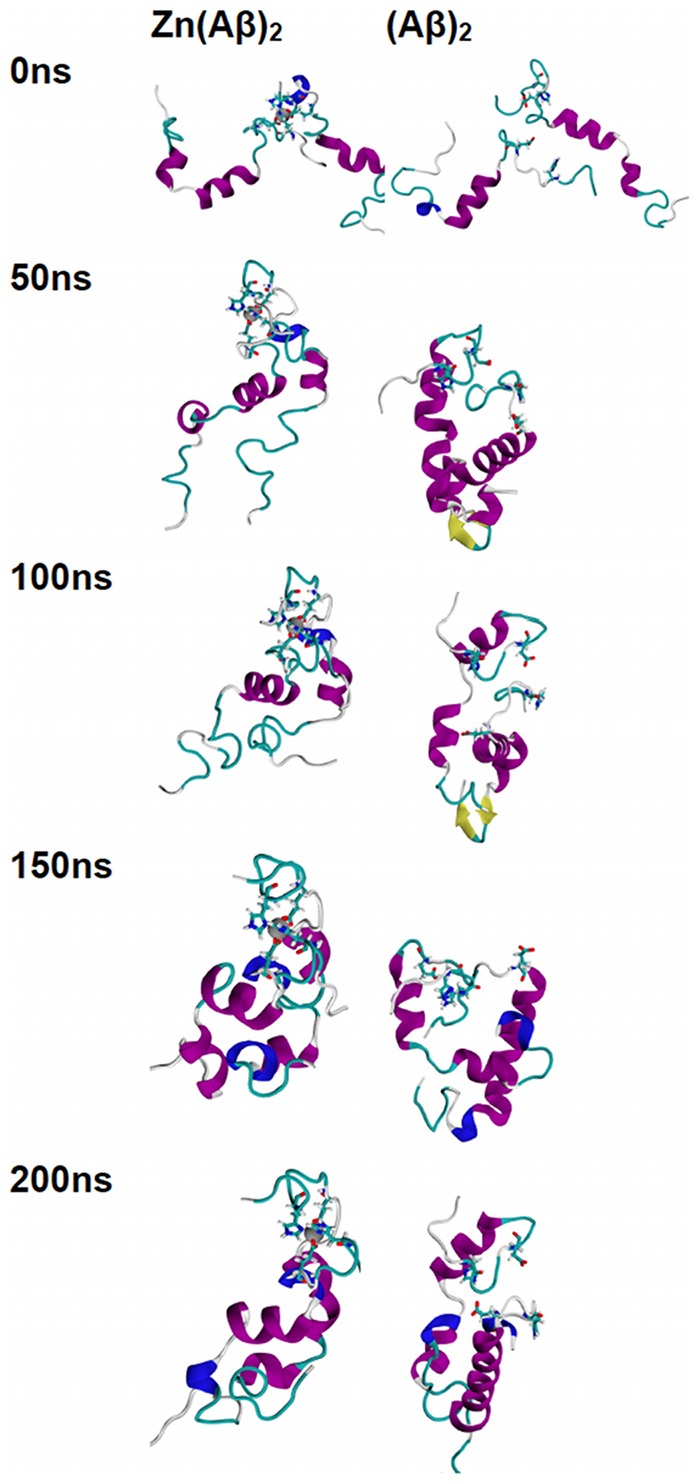
Trajectories of Simulations of Zn(Aβ)_2_ (Glu11 and His6) and Control. Structures of Zn(Aβ)_2_ (where Zn bridges at Glu11 and His6), and the control (Aβ)_2_ at 0 ns, 50 ns, 100 ns, 150 ns and 200 ns are shown. The peptides have the same initial structure in the Zn(Aβ)_2_ complex and the control simulation. Gray spheres are zinc atoms. Zn-binding residues including Glu11 and His6 are shown in licorice, where red, blue and cyan colors shows the atoms and bonds for oxygen, nitrogen and carbon respectively. Peptide backbones are shown in cartoon style where cyan indicates turn, white is coil, purple is alpha-helix, blue is 3_10_ helix, and yellow is β-sheet. All simulations were performed in explicit water. Water molecules are not shown for clarity. Each is peptide oriented with the N-terminus at the top and C-terminus towards the bottom.

**Figure 3:**
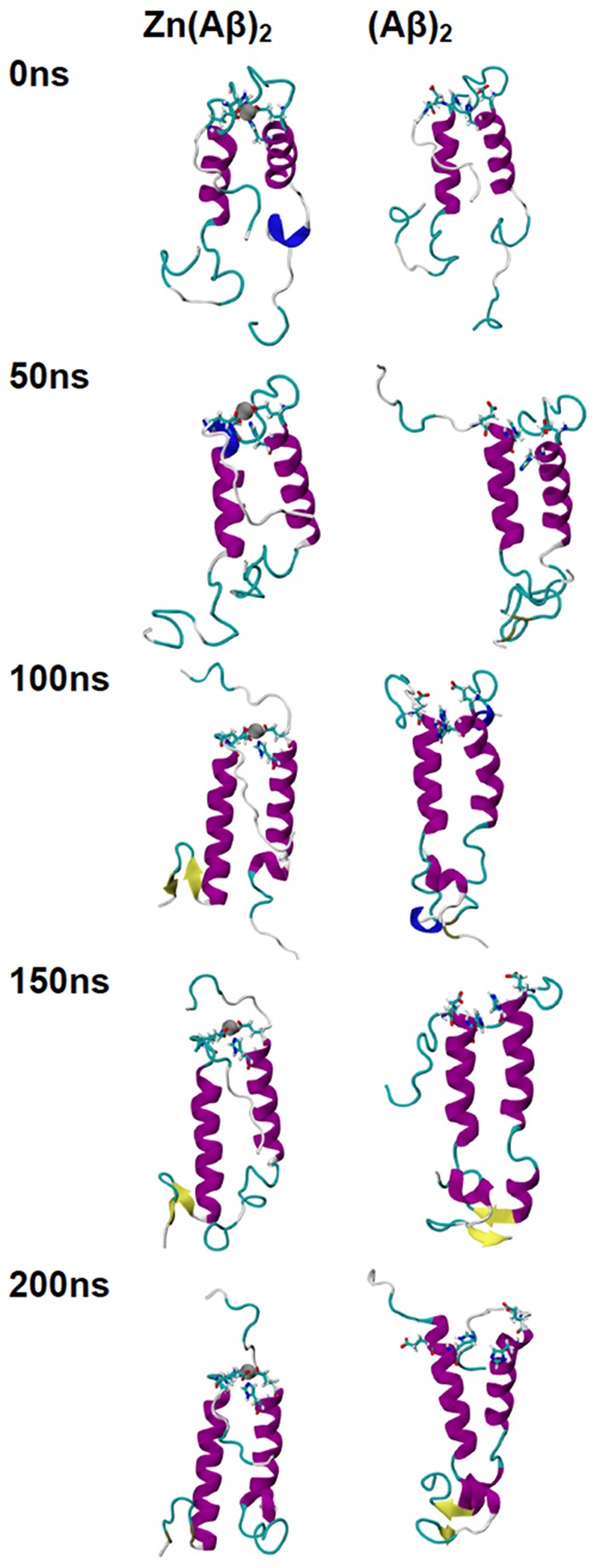
Trajectories of Simulations of Zn(Aβ)_2_ (Glu11 & His13) and Control. Structures of Zn(Aβ)_2_ (where Zn bridges at Glu11 and His13), and the control (Aβ)_2_ at 0 ns, 50 ns, 100 ns, 150 ns and 200 ns are shown. The peptides have the same initial structure in the Zn(Aβ)_2_ complex and the control simulation. Gray spheres are zinc atoms. Zn-binding residues including Glu11 and His13 are shown in licorice, where red, blue and cyan colors shows the atoms and bonds for oxygen, nitrogen and carbon respectively. Peptide backbones are shown in cartoon style where cyan indicates turn, white is coil, purple is alpha-helix, blue is 3_10_ helix, and yellow is β-sheet. All simulations were performed in explicit water. Water molecules are not shown for clarity. Each is peptide oriented with the N-terminus at the top and C-terminus towards the bottom.

**Figure 4:**
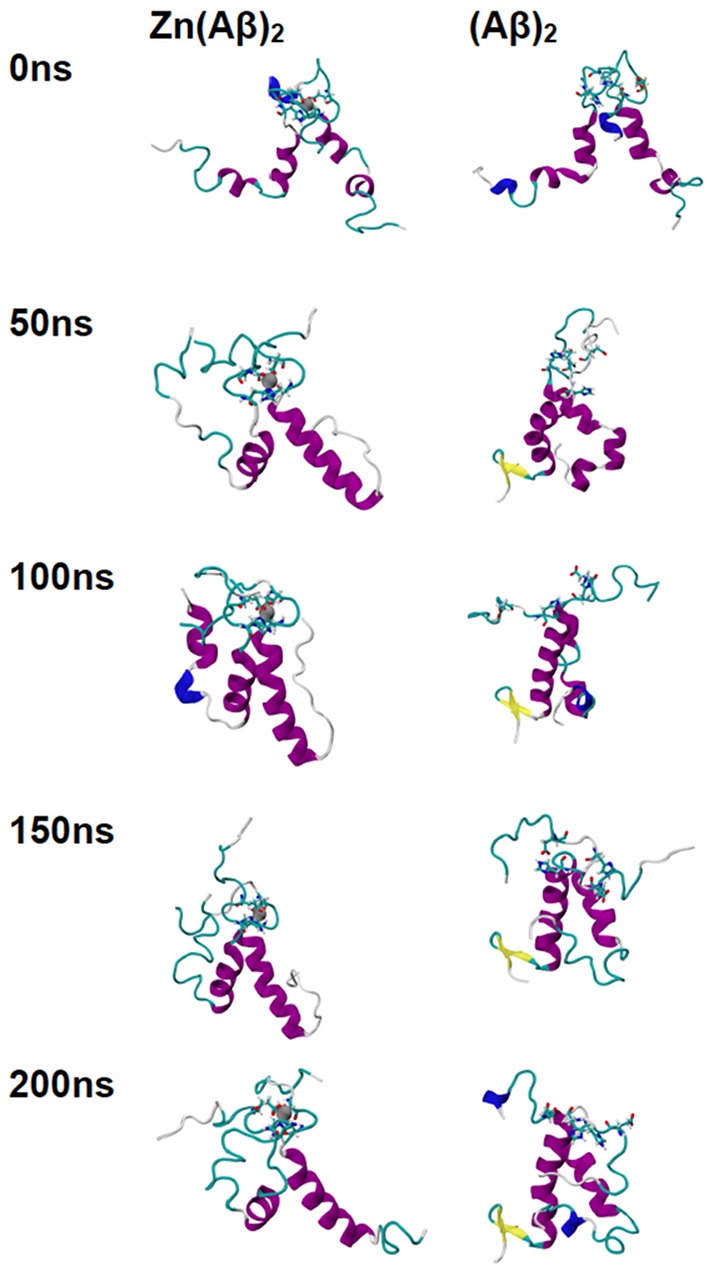
Trajectories of Simulations of Zn(Aβ)_2_ (Glu11 & His14) and Control. Structures of Zn(Aβ)_2_ (where Zn bridges at Glu11 and His14), and the control (Aβ)_2_ at 0 ns, 50 ns, 100 ns, 150 ns and 200 ns are shown. The peptides have the same initial structure in the Zn(Aβ)_2_ complex and the control simulation. Gray spheres are zinc atoms. Zn-binding residues including Glu11 and His14 are shown in licorice, where red, blue and cyan colors shows the atoms and bonds for oxygen, nitrogen and carbon respectively. Peptide backbones are shown in cartoon style where cyan indicates turn, white is coil, purple is alpha-helix, blue is 3_10_ helix, and yellow is β-sheet. All simulations were performed in explicit water. Water molecules are not shown for clarity. Each is peptide oriented with the N-terminus at the top and C-terminus towards the bottom.

**Figure 5:**
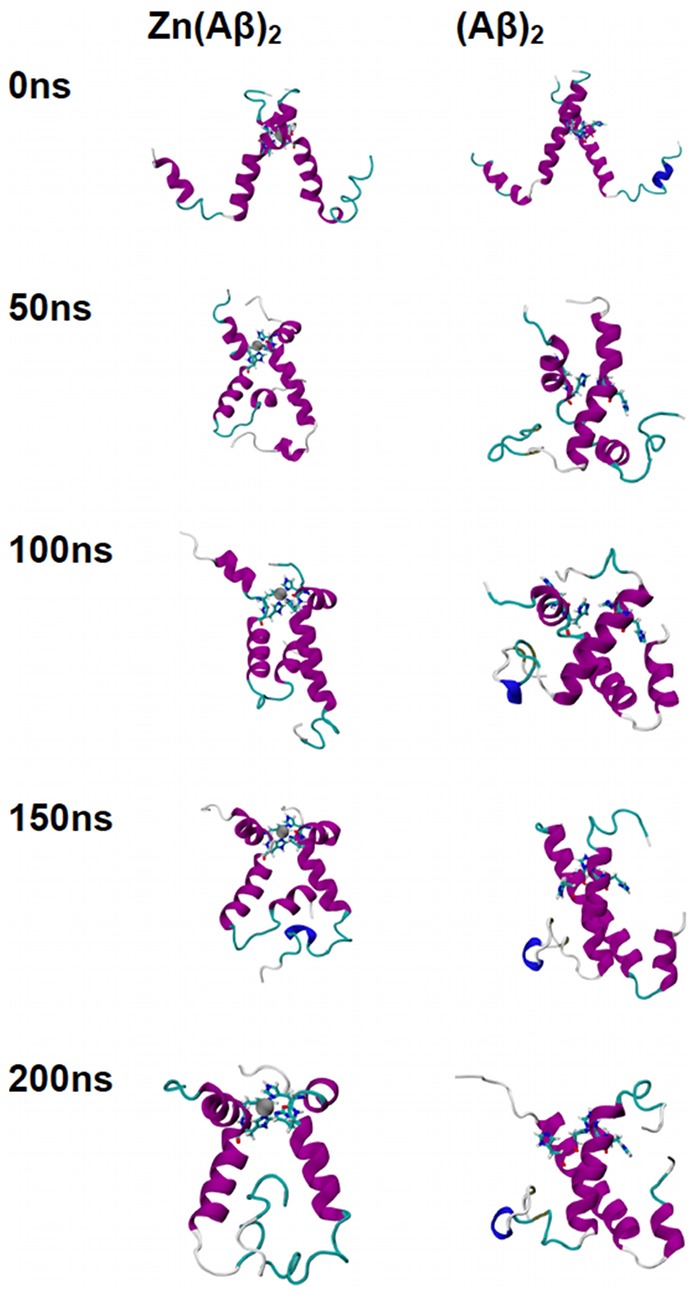
Trajectories of Simulations of Zn(Aβ)_2_ (His13 & His14) and Control. Structures of Zn(Aβ)_2_ (where Zn bridges at His13 and His14), and the control (Aβ)_2_ at 0 ns, 50 ns, 100 ns, 150 ns and 200 ns are shown. The peptides have the same initial structure in the Zn(Aβ)_2_ complex and the control simulation. Gray spheres are zinc atoms. Zn-binding residues including His13 and His14 are shown in licorice, where red, blue and cyan colors shows the atoms and bonds for oxygen, nitrogen and carbon respectively. Peptide backbones are shown in cartoon style where cyan indicates turn, white is coil, purple is alpha-helix, blue is 3_10_ helix, and yellow is β-sheet. All simulations were performed in explicit water. Water molecules are not shown for clarity. Each is peptide oriented with the N-terminus at the top and C-terminus towards the bottom.

The trajectories of all the species over the 200 ns of simulation time are shown in Figures 1, 2, 3, 4, and 5. For Zn(Aβ) (Figure 1), Zn binding disturbed the helix in the N-terminus, and those areas remained random coil throughout the simulation, whereas the controls, which model the behavior of Aβ after Zn dissociates, regain helical structure in the N-terminus. For the Zn(Aβ)_2_ complexes with Zn bridging at His 6 and Glu11 (Figure 2), the zinc forced the two peptide chains to adopt a perpendicular orientation to accommodate the local Zn-binding residues. In the initial structure, the Zn binding brought the N-termini of the two peptides together, whereas the C-termini remained separated from each other. However, after equilibration, the C-termini of the peptide chains had moved and were in contact. For the control system, which started with the same initial structure as the Zn(Aβ)_2_ (His6/Glu11), both the N- and C-termini of the peptides moved closer to each other despite the absence of a Zn linkage. A stable intra-chain β-sheet was formed at the C-terminus of the control simulation after 50 ns. For Aβ dimers with Zn bridging at Glu11 and His13 (Figure 3), the peptide chains remained parallel due to the constraints imposed by the Zn-binding residues. Both peptides remained in contact with each other and maintained helical structures despite Zn-binding. In both the Zn-bound and the control simulations, the peptides acquired β-sheet structures at their respective C-termini after 100 ns. In the Zn-bound simulation, the peptides formed a single intra-chain β-sheet, while in the control simulation there was an inter-chain β-sheet. For the peptide dimers with Zn bridging at Glu11 and His14 (Figure 4), the peptides are forced into a 45° angle with respect to each other due to constraints imposed by binding to Zn and regional conformations from the Aβ(1-16) NMR structure. The peptides in the Zn-bound and control simulations both lost helix at their respective N-termini. β-sheet formation was observed at the C-terminus of one of the peptides in the control simulations. For Aβ dimers with Zn bridging at His13 and His14 (Figure 5), the two peptide chains are parallel. The peptides in the Zn-bound system lost helix at the N- and C-termini, while the peptides in the control system regained helix during the simulations. In general, once zinc binds and bridges two peptides, those peptides remain in contact even after zinc dissociates and leaves the binding site. Peptides that are not arranged in a parallel configuration tend to lose helical structure while parallel peptide configurations stabilize the helix. All β-sheet structures were observed at the C-terminus of Aβ, which suggests that this region initiates changes in secondary structure that lead to peptide aggregation.

Analysis of root mean square deviations of each simulation (Figure S1), shows that all systems achieve at least 80 ns of equilibrated dynamics. For the monomers, both the control and the Zn-bound Aβ simulations reach equilibrium after 120 ns. For the dimers, the four Zn-bound simulations and their respective control simulations all reach equilibrium before 100 ns.

**Figure 6:**
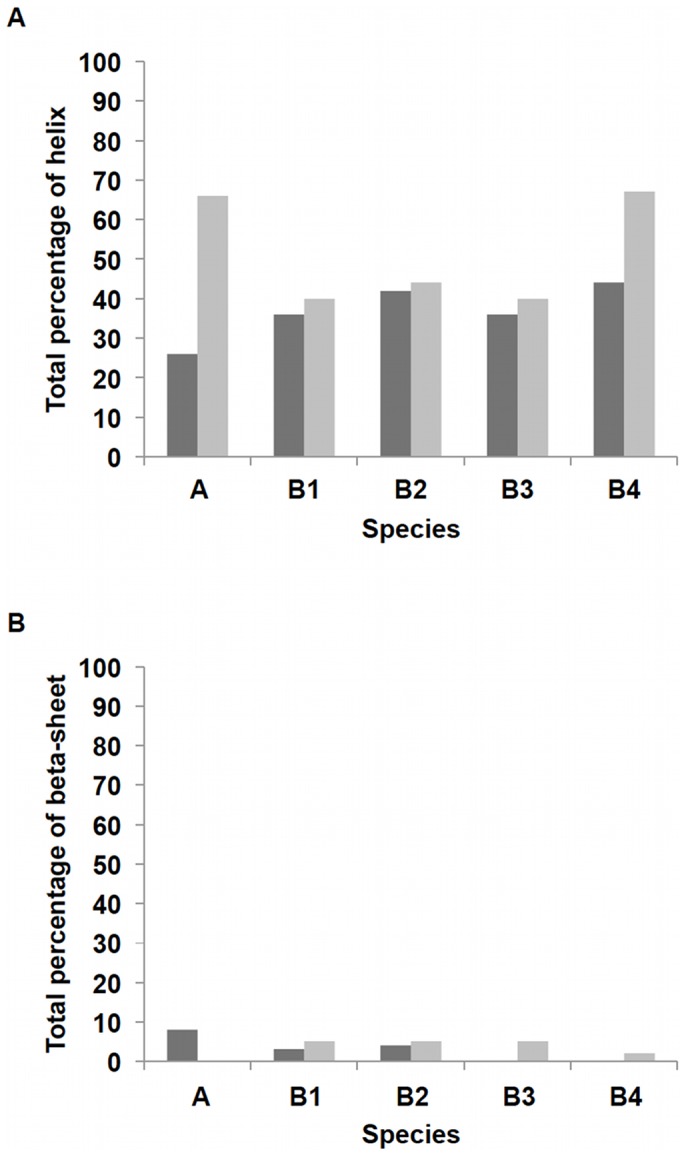
Total Percentage of Secondary Structure. Percentages of helix and β-sheet for each chain after equilibrium are shown. The peptides in each Zn-bound complex and their corresponding controls have the same initial structures. **A** shows Zn(Aβ) (where Zn binds at Glu11 His6, 13 14) and control. **B1** shows Zn(Aβ)_2_ (where Zn bridges at Glu11 and His6) and control. **B2** shows Zn(Aβ)_2_ (where Zn bridges at Glu11 and His13) and control. **B3** shows Zn(Aβ)_2_ (where Zn bridges at His13 and His 14) and control. **B4** shows Zn(Aβ)_2_ (where Zn bridges at Glu11 and His14) and control. Dark gray lines are for Zn-bound complexes. Gray lines are for controls.

**Figure 7:**
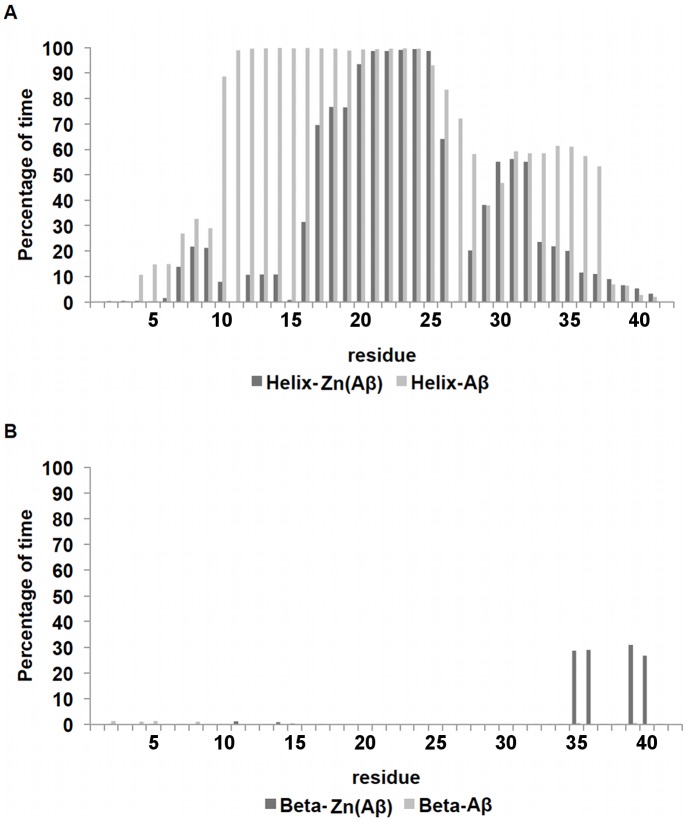
Per-Residue Secondary Structure of Zn(Aβ) and Control. The percentage of time (after equilibration) peptide residues adopt helix or β-sheet structure is shown. (A) shows the per-residue helix. (B) shows the per-residue β-sheet. Dark gray lines are Zn(Aβ) where Zn binds at Glu11 His6, 13 14. Gray lines are the control. The peptide has the same initial structure in the Zn(Aβ) complex and the control simulation.

The total percentage of secondary structure composition (Figure 6) shows that Zn-binding results in a lower total helix percentage after equilibration, but increased β-sheet percentage at the C-termini of various species. Analysis of the per-residue-helicity for each peptide (Figure 7 and Figure S3), shows that for the monomeric species, Zn binding caused the loss of helix structure from residue 6 to residue 14, which extended the random coil structure from residue 1 to residue 20. On the other hand, the control peptide remains mostly helical from residue 6 to the C-terminus, with the exception of residues N_27_KGA_30_ and the last three residues. Both systems possess the loop region N_27_KGA_30_ which is the same loop found in the Aβ fibril structure [44]. For the dimeric complexes (Figure S3), the N- and C-termini show a decrease of helical structures in general. However, the total percentage of helix at the C-terminus only showed a modest decrease. Interestingly, for all simulations of Zn(Aβ)_2_, the decrease in helix occurred near residue Asn27 which is in the loop that is vital to the formation of β-sheet structure in the Aβ fibrils. Similarly, the formation of β-sheet among all the dimer simulations occurred in the same region, G_33_LMVGGVV_40_, regardless of whether it was inter-chain or intra-chain β-sheet, which suggests a common mechanism for the secondary structure transition.

**Figure 8:**
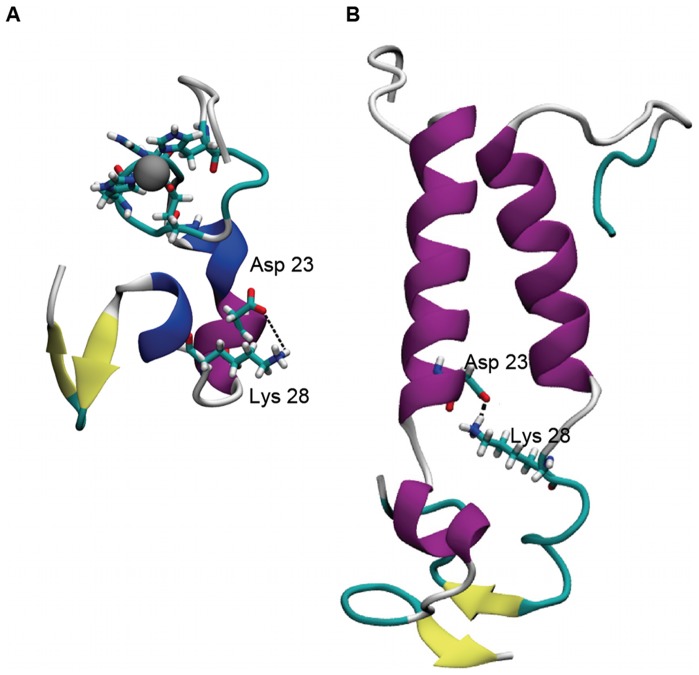
Intra- and Inter-chain Salt Bridge (Asp23-Lys28). Structure **A** shows an intra-chain salt bridge between Asp23 and Lys28 in Zn(Aβ) at 200 ns. Structure **B** shows an inter-chain salt bridge between Asp23 and Lys28 in Zn(Aβ)_2_ with Zn bridging at Glu11 and His13 at 200 ns. Gray spheres are Zn. Zn-binding residues (**A**: Glu11, His6, 13, 14; **B**:Glu11 and His13) are represented in licorice where red, blue and cyan represent oxygen, nitrogen and carbon respectively. Peptide backbones are shown in cartoon style where cyan indicates turn, white is coil, purple is alpha-helix, blue is 3_10_ helix, and yellow is β-sheet.

The disruption of the helix structures in the N_27_KGA_30_ region facilitates the formation of the Asp23-Lys28 salt bridge (Figure 8) that stabilizes the Aβ fibrils. Hence, we analyzed this salt bridge over time and the results are shown in Table 1. For the monomeric species, the Zn-bound Aβ showed a constant intra-chain salt bridge Asp23-Lys28 formation over 48.1% of time whereas in the control species the salt bridge did not form. Zn binding promoted the formation of the salt bridge. For the Zn(Aβ)_2_ complexes, various intra-chain and inter-chain Asp23-Lys28 salt bridges were observed for both Zn(Aβ)_2_ and (Aβ)_2_ species (Table 1). In general, the intra-chain salt bridge helps maintain the loop N_27_KGA_30._ The inter-chain salt bridges enhance the peptide-peptide interactions and keep both peptides in contact even without the Zn linkage.

**Figure 9:**
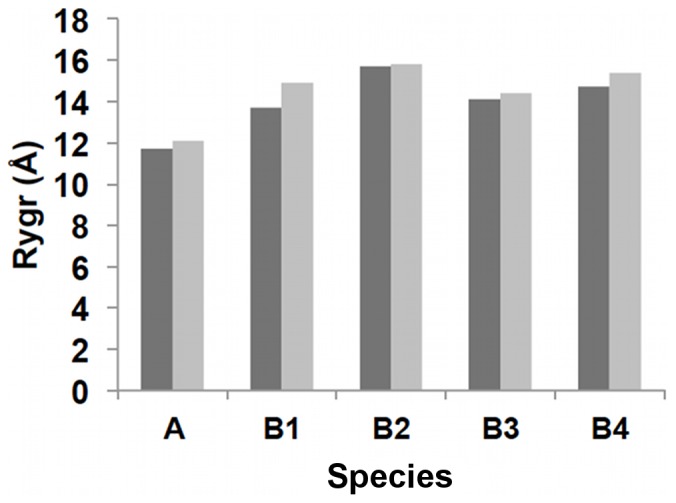
Average Equilibrium Radius of Gyration (Rygr) (Å). Average equilibrium radii of gyration are shown. **A** shows Zn(Aβ) (where Zn binds at Glu11 His6, 13 14) and control. **B1** shows Zn(Aβ)_2_ (where Zn bridges at Glu11 and His6) and control. **B2** shows Zn(Aβ)_2_ (where Zn bridges at Glu11 and His13) and control. **B3** shows Zn(Aβ)_2_ (where Zn bridges at His13 and His 14) and control. **B4** shows Zn(Aβ)_2_ (where Zn bridges at Glu11 and His14) and control. Dark gray lines are for Zn-bound complexes. Gray lines are for controls.

The radius of gyration analysis reveals the general compactness of the peptide complexes. Comparison between Zn-bound and control species (Figure 9) showed that Zn binding results in a slightly more compact conformation during equilibrium. For proteins with the same number of residues, those with α helix structures have the largest radii of gyration [51]. In general, decreases in the radius of gyration reflect the loss of helix.

## Discussion

Aβ peptide is one of the intrinsically disordered proteins that have no native tertiary structure. Therefore, its conformation largely depends on the complex and often transient interactions between multiple environmental factors. For example, the free soluble Aβ peptide in water/hexafluoroisopropanol (HFIP) 4∶1 solution [37] is mostly helical whereas the fibril Aβ is dominated by β-sheet [38]. The transformation of helix to β-sheet needs to go through the unfolding of helical turns (to coil) and refolding (to β-sheet). Previous experiments showed that the ratio of zinc: Aβ in senile plaques ranges from 1∶1 to 1∶200 [52]. The Zn(Aβ) simulation results suggests that if zinc: Aβ has a 1∶1 ratio, zinc binding to Aβ monomer can disrupt the helix structure not only on the zinc binding regions but also in both the N- and C-terminus. Kinetically, the ligand exchange rate of zinc is greater than that of most other first row transition metals [53]. Therefore, zinc is constantly associating to and dissociating from Aβ. The results of the Zn-bound and control Aβ dimer models demonstrate the influence of Zn binding and its extended effects on the peptide conformation after dissociation. In this work, Zn was covalently bound to Aβ peptides to simulate a sufficient concentration of Zn such that Aβ peptides would be effectively coordinated for enough time to form Aβ-Aβ interactions. Once these interactions form, even if Zn(II) subsequently dissociates, conformational changes in the C-terminus can still occur. Although the peptides in the Zn-bound and the control Aβ dimer simulations have similar behavior, they do differ in the nature of the salt-bridges that they form. In particular, the Zn-bound dimer simulations show that formation of the critical D23/K28 intra-chain salt-bridge occurs and persists with the exception of the Zn(Aβ)_2_ (His13/His14) case. The formation and stabilization of the intra-chain D23/K28 salt-bridge has been shown to be important in accelerating the fibrillation of Aβ(1-40) [54]. Nevertheless, none of the Zn-bound dimer simulations shows the formation of inter-chain D23/K28 salt-bridges. Interestingly, inter-chain D23/K28 salt-bridges do form in the simulations for controls for the Zn(Aβ)_2_ (Glu11/His13) and (Glu11/His14) simulations. It worth noting that inter-chain D23/K28 salt-bridges have been observed by NMR for the Aβ(1-40) fibrils [55]. This suggests that zinc accelerates fibrillation of Aβ at least in part as a result of its lability. By binding and dissociating from Aβ, Zn alternately promotes the formation of intra- and inter-chain D23/K28 salt-bridges, respectively.

In all of the simulations, β-sheet formation is observed exclusively at the C-terminus, G_31_LMVGGVVI_42_, which suggests that the peptide's secondary structure transition starts in this region. This result is in agreement with a recent study on the assembly processes of Aβ(1-42) and Aβ(1-40) [56], which showed that β-sheet structures were observed in the G_31_LMVGGVVI_42_ region for Aβ(1-42). In addition, it was shown that C-terminal β-sheet formation is positively correlated to the increased toxicity of Aβ peptide species.

Our results suggests that a sufficient concentration Zn(II) ions can promote the oligomerization of Aβ by disrupting the helical structures in the N-terminus, inducing β-sheet formation in the C-terminus and promoting the formation of both intra- and inter-chain salt-bridges. These results agree with the observation that zinc promotes rapid aggregation with both parallel and non-parallel assembly and form non-fibril aggregated species [41]. The simulations in this work shows how Zn binding to Aβ can promote and accelerate the helix-to-β-sheet transition in this region, which might explain how zinc enhances the toxicity of Aβ.

## Supporting Information

Figure S1 **Root Mean Square Deviations (Cα only) for each Simulation.** For each simulation, root mean square deviation (RMSD) is calculated for the entire trajectory using the first trajectory as the reference. Gray lines are Zn-bound complexes. Dark gray lines are controls.
(DOCX)

Figure S2 **Root Mean Square Fluctuation (Cα only) of each Simulation.** For each simulation, root mean square fluctuation (RMSF) is calculated for the equilibrated portion of the simulation (last 80 ns). Gray lines are Zn-bound complexes. Dark gray lines are controls.
(DOCX)

Figure S3 **Per-Residue Helix and Beta-sheet Content for each Zn(Aβ)_2_.** All per-residue secondary structure results are average values of all dimeric species over three runs for each simulation. Gray lines are Zn-bound complexes. Dark gray lines are controls.
(DOCX)

Table S1 **Force Constants for Zn and Coordinating Residues.** List of force constants between zinc and the coordinating atoms obtained from DFT calculations at the B3LYP/6-31+G* level that were added to the CHARMM 22/CMAP force field parameters. The force-field potential energy as found in CHARMM22 is as follows: BONDS: V(bond) = K_b_(b−b_0_)^2^, K_b_: kcal/mole/A^2^, b_0_; ANGLES: V(angle) = K_θ_(θ−θ_0_)^2^, K_θ_: kcal/mole/rad^2^, θ_0_: degrees; DIHEDRALS: V(dihedral) = K_χ_(1+cos(n(χ)−γ)), K_χ_: kcal/mole, n: multiplicity, γ: degrees.
(DOCX)
