# Supplementary material for: Molecular Dynamics Study of Zn(Aβ) and Zn(Aβ)2
Source: PLoS One. 2013 Sep 27;8(9):e70681. doi: 10.1371/journal.pone.0070681 (PMC3785486; doi:10.1371/journal.pone.0070681)
Supplement: Figure S1 — Root Mean Square Deviations (Cα only) for each Simulation. For each simulation, root mean square deviation (RMSD) is calculated for the entire trajectory using the first trajectory as the reference. Gray lines are Zn-bound complexes. Dark gray lines are controls. (DOCX) [file pone.0070681.s001.docx]

**Figure S1. Root Mean Square Deviations (Cα only) for each Simulation**

A. Monomer, Zn binding at His6, 13, 14 and Glu11

B1. Dimer, Zn bridging at His6 and Glu11

B2. Dimer, Zn bridging at Glu11 and His13


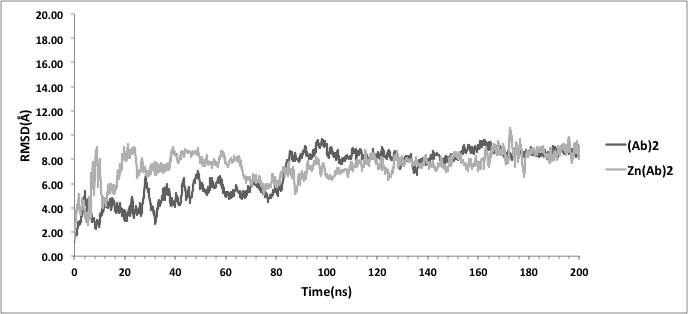


B3. Dimer, Zn bridging at Glu11 and His14

B4. Dimer, Zn bridging at His13 and His14

For each simulation, root mean square deviation (RMSD) is calculated for the entire trajectory using the first trajectory as the reference. Gray lines are Zn-bound complexes. Dark gray lines are controls.
