# Supplementary material for: Molecular Dynamics Study of Zn(Aβ) and Zn(Aβ)2
Source: PLoS One. 2013 Sep 27;8(9):e70681. doi: 10.1371/journal.pone.0070681 (PMC3785486; doi:10.1371/journal.pone.0070681)
Supplement: Figure S2 — Root Mean Square Fluctuation (Cα only) of each Simulation. For each simulation, root mean square fluctuation (RMSF) is calculated for the equilibrated portion of the simulation (last 80 ns). Gray lines are Zn-bound complexes. Dark gray lines are controls. (DOCX) [file pone.0070681.s002.docx]

**Figure S2. Root Mean Square Fluctuation (Cα only) of each Simulation**

A. Monomer, Zn binding at His6, 13, 14 and Glu11

B1. Dimer, Zn bridging at His6 and Glu11

B2. Dimer, Zn bridging at Glu11 and His13

B3. Dimer, Zn bridging at Glu11 and His14

B4. Dimer, Zn bridging at His13 and His14

For each simulation, root mean square fluctuation (RMSF) is calculated for the equilibrated portion of the simulation (last 80ns). Gray lines are Zn-bound complexes. Dark gray lines are controls.
