# Supplementary material for: Molecular Dynamics Study of Zn(Aβ) and Zn(Aβ)2
Source: PLoS One. 2013 Sep 27;8(9):e70681. doi: 10.1371/journal.pone.0070681 (PMC3785486; doi:10.1371/journal.pone.0070681)
Supplement: Figure S3 — Per-Residue Helix and Beta-sheet Content for each Zn(Aβ)2. All per-residue secondary structure results are average values of all dimeric species over three runs for each simulation. Gray lines are Zn-bound complexes. Dark gray lines are controls. (DOCX) [file pone.0070681.s003.docx]

**Figure S3. Per-Residue Helix and Beta-sheet Content for each Zn(Aβ)_2_.**

All per-residue secondary structure results are average values of all dimeric species over three runs for each simulation. Gray lines are Zn-bound complexes. Dark gray lines are controls.
