# Supplementary material for: Molecular Dynamics Study of Zn(Aβ) and Zn(Aβ)2
Source: PLoS One. 2013 Sep 27;8(9):e70681. doi: 10.1371/journal.pone.0070681 (PMC3785486; doi:10.1371/journal.pone.0070681)
Supplement: Table S1 — Force Constants for Zn and Coordinating Residues. List of force constants between zinc and the coordinating atoms obtained from DFT calculations at the B3LYP/6-31+G* level that were added to the CHARMM 22/CMAP force field parameters. The force-field potential energy as found in CHARMM22 is as follows: BONDS: V(bond) = Kb(b−b0)2, Kb: kcal/mole/A2, b0; ANGLES: V(angle) = Kθ(θ−θ0)2, Kθ: kcal/mole/rad2, θ0: degrees; DIHEDRALS: V(dihedral) = Kχ(1+cos(n(χ)−γ)), Kχ: kcal/mole, n: multiplicity, γ: degrees. (DOCX) [file pone.0070681.s004.docx]

**Table S1. Force Constants for Zn and Coordinating Residues**

A. [Zn(Im)_3_Ac]^+^

| Bond | K_b_ (kcal/mol/Å^2^) | b_0_ (Å) | |
| --- | --- | --- | --- |
| Zn-O  Zn-N1  Zn-N2  Zn-N3 | 129.267  86.350  86.350  74.367 | 1.9741  2.0532  2.0532  2.0838 | |
| Angle | K_θ_ (kcal/mol/rad^2^) | θ_0_ (degree) | |
| N1-Zn-O  N1-Zn-N2  N1-Zn-N3  O-Zn-N2  O-Zn-N3  N2-Zn-N3  Zn-N1-C1  Zn-N1-C2  Zn-O-C  Zn-N2-C3  Zn-N2-C4  Zn-N3-C5  Zn-N3-C6 | 5.89  3.311  5.39  5.964  4.833  5.403  19.682  22.451  73.034  19.597  22.204  17.52  15.675 | 115.25  112.09  107.43  115.25  97.77  107.43  130.52  122.75  105.57  130.52  122.75  119.15  134.19 | |
| Dihedral | K_χ_ (kcal/mole) | n | γ (degree) |
| O-Zn-N1-C1  O-Zn-N1-C2  N2-Zn-N1-C1  N2-Zn-N1-C2  N3-Zn-N1-C1  N3-Zn-N1-C2  N1-Zn-O-C  N2-Zn-O-C  N3-Zn-O-C  N1-Zn-N2-C3  N1-Zn-N2-C4  O-Zn-N2-C3  O-Zn-N2-C4  N3-Zn-N2-C3  N3-Zn-N2-C4  N1-Zn-N3-C5  N1-Zn-N3-C6  O-Zn-N3-C5  O-Zn-N3-C6  N2-Zn-N3-C5  N2-Zn-N3-C6 | 0.066  0.075  0.057  0.06  0.043  0.046  0.898  0.89  0.553  0.058  0.061  0.069  0.078  0.045  0.049  0.04  0.041  0.064  0.06  0.041  0.041 | 6  6  6  6  6  6  3  3  3  6  6  6  6  6  6  6  6  6  6  6  6 | 122.07  -55.31  -103.48  79.14  14.32  -163.06  66.5  -66.51  179.99  103.47  -79.15  -122.07  55.31  -14.32  163.06  119.62  -60.38  0.01  -179.99  -119.6  60.4 |

B. [Zn(Im)_2_(Ac)_2_]

| Bond | K_b_ (kcal/mol/Å^2^) | b_0_ (Å) | |
| --- | --- | --- | --- |
| Zn-N1  Zn-O1  Zn-N2  Zn-O2 | 78.856  115.4  79.065  122.969 | 2.0726  1.9727  2.0724  1.9535 | |
| Angle | K_θ_ (kcal/mol/rad^2^) | θ_0_ (degree) | |
| N1-Zn-O1  N1-Zn-N2  N1-Zn-O2  N2-Zn-O1  N2-Zn-O2  O1-Zn-O2  Zn-N1-C1  Zn-N1-C2  Zn-O1-C3  Zn-N2-C4  Zn-N2-C5  Zn-O2-C6 | 9.785  4.619  7.71  9.957  5.36  7.834  21.069  22.047  55.627  21.411  22.616  42.712 | 109.52  104.23  113.62  109.56  106.28  113.6  127.6  125.19  121.02  127.58  125.21  115.96 | |
| Dihedral | K_χ_ (kcal/mole) | n | γ (degree) |
| O1-Zn-N1-C1  O1-Zn-N1-C2  N2-Zn-N1-C1  N2-Zn-N1-C2  O2-Zn-N1-C1  O2-Zn-N1-C2  N1-Zn-O1-C3  O2-Zn-O1-C3  N2-Zn-O1-C3  N1-Zn-N2-C4  N1-Zn-N2-C5  O1-Zn-N2-C4  O1-Zn-N2-C5  O2-Zn-N2-C4  O2-Zn-N2-C5  N1-Zn-O2-C6  O1-Zn-O2-C6  N2-Zn-O2-C6 | 0.089  0.094  0.101  0.102  0.063  0.062  0.652  0.663  0.577  0.098  0.099  0.087  0.092  0.061  0.06  0.416  0.493  0.431 | 6  6  6  6  6  6  3  3  3  6  6  6  6  6  6  3  3  3 | 149.71  -29.85  -93.13  87.3  31.04  -148.52  56.91  -56.84  -179.96  93.08  -87.39  -149.8  29.74  -31.11  148.42  -59.44  -179.96  59.5 |

C. [Zn(Im)_4_]^2+^

| Bond | K_b_ (kcal/mol/Å^2^) | b_0_ (Å) | |
| --- | --- | --- | --- |
| Zn-N1  Zn-N2  Zn-N3  Zn-N4 | 89.977  90.118  90.227  90.123 | 2.0387  2.0387  2.0386  2.0386 | |
| Angle | K_θ_ (kcal/mol/rad^2^) | θ_0_ (degree) | |
| N1-Zn-N2  N1-Zn-N3  N1-Zn-N4  N2-Zn-N3  N2-Zn-N4  N3-Zn-N4  Zn-N1-C1  Zn-N1-C2  Zn-N2-C3  Zn-N2-C4  Zn-N3-C5  Zn-N3-C6  Zn-N4-C7  Zn-N4-C8 | 3.18  2.724  2.929  2.983  2.743  3.346  16.291  15.638  16.543  15.796  15.821  15.549  16.07  15.735 | 109.09  111.72  109.46  109.47  111.74  105.34  125.28  128.27  125.28  128.26  125.14  128.43  125.14  128.43 | |
| Dihedral | K_χ_ (kcal/mole) | n | γ (degree) |
| N2-Zn-N1-C1  N2-Zn-N1-C2  N3-Zn-N1-C1  N3-Zn-N1-C2  N4-Zn-N1-C1  N4-Zn-N1-C2  N1-Zn-N2-C3  N1-Zn-N2-C4  N3-Zn-N2-C3  N3-Zn-N2-C4  N4-Zn-N2-C3  N4-Zn-N2-C4  N1-Zn-N3-C5  N1-Zn-N3-C6  N2-Zn-N3-C5  N2-Zn-N3-C6  N4-Zn-N3-C5  N4-Zn-N3-C6  N1-Zn-N4-C7  N1-Zn-N4-C8  N2-Zn-N4-C7  N2-Zn-N4-C8  N3-Zn-N4-C7  N3-Zn-N4-C8 | 0.031  0.031  0.036  0.037  0.033  0.035  0.031  0.031  0.033  0.034  0.034  0.035  0.034  0.031  0.033  0.031  0.033  0.033  0.034  0.033  0.035  0.032  0.035  0.034 | 6  6  6  6  6  6  6  6  6  6  6  6  6  6  6  6  6  6  6  6  6  6  6  6 | 138.37  -41.97  -100.46  79.2  15.82  -164.52  138.43  -41.87  15.9  -164.4  -100.4  79.3  163.06  -17  -75.99  103.95  44.3  -135.76  -76.28  103.71  162.77  -17.25  43.99  -136.03 |

List of force constants between zinc and the coordinating atoms obtained from DFT calculations at the B3LYP/6-31+G* level that were added to the CHARMM 22/CMAP force field parameters. The force-field potential energy as found in CHARMM22 is as follows: BONDS: V(bond) = K_b_(b - b_0_)^2^, K_b_: kcal/mole/A^2^, b_0_; ANGLES: V(angle) = K_θ_(θ - θ_0_)^2^, K_θ_: kcal/mole/rad^2^, θ_0_: degrees; DIHEDRALS: V(dihedral) = K_χ_(1 + cos(n(χ) - γ)), K_χ_: kcal/mole, n: multiplicity, γ: degrees
